# Metagenomic Analysis of the Bioremediation of Diesel-Contaminated Canadian High Arctic Soils

**DOI:** 10.1371/journal.pone.0030058

**Published:** 2012-01-11

**Authors:** Etienne Yergeau, Sylvie Sanschagrin, Danielle Beaumier, Charles W. Greer

**Affiliations:** National Research Council Canada, Biotechnology Research Institute, Montreal, Quebec, Canada; Argonne National Laboratory, United States of America

## Abstract

As human activity in the Arctic increases, so does the risk of hydrocarbon pollution events. On site bioremediation of contaminated soil is the only feasible clean up solution in these remote areas, but degradation rates vary widely between bioremediation treatments. Most previous studies have focused on the feasibility of on site clean-up and very little attention has been given to the microbial and functional communities involved and their ecology. Here, we ask the question: which microorganisms and functional genes are abundant and active during hydrocarbon degradation at cold temperature? To answer this question, we sequenced the soil metagenome of an ongoing bioremediation project in Alert, Canada through a time course. We also used reverse-transcriptase real-time PCR (RT-qPCR) to quantify the expression of several hydrocarbon-degrading genes. *Pseudomonas* species appeared as the most abundant organisms in Alert soils right after contamination with diesel and excavation (t = 0) and one month after the start of the bioremediation treatment (t = 1m), when degradation rates were at their highest, but decreased after one year (t = 1y), when residual soil hydrocarbons were almost depleted. This trend was also reflected in hydrocarbon degrading genes, which were mainly affiliated with *Gammaproteobacteria* at t = 0 and t = 1m and with *Alphaproteobacteria* and *Actinobacteria* at t = 1y. RT-qPCR assays confirmed that *Pseudomonas* and *Rhodococcus* species actively expressed hydrocarbon degradation genes in Arctic biopile soils. Taken together, these results indicated that biopile treatment leads to major shifts in soil microbial communities, favoring aerobic bacteria that can degrade hydrocarbons.

## Introduction

With the ongoing global rise in temperature, there is increased political, scientific and economic interest in the Arctic regions. The resulting increased activity in the Arctic is raising the risk of accidental hydrocarbon spills as hydrocarbons, like diesel, are used for heating, transportation and electricity. In remote areas, like the Canadian high Arctic, on-site bioremediation is the only feasible clean-up option for hydrocarbon spills. A number of studies have demonstrated that microorganisms, in particular bacteria, are capable of degrading hydrocarbons at the extreme temperatures typically encountered in polar and alpine environments [1]–[6]. In addition to the low temperature, limiting factors for the bioremediation of contaminated soils in polar environments are related to the availability of other essential nutrients (mainly N and P) and the low levels of available water. One approach that has proven successful in polar environments is to fertilize the soils to stimulate the activity of indigenous hydrocarbon-degrading microorganisms [2], [3], [5]–[12]. These indigenous microorganisms have the advantage of being ideally adapted to the environmental conditions prevailing at the site to be remediated. However, bioremediation experiments often show large, unexplained differences in hydrocarbon degradation rates and, thus, in the time required to achieve complete remediation.

A recent study from our group identified some of the factors influencing the microbial community structure, the expression of genes involved in bioremediation and the subsequent rate of hydrocarbon mineralization [13]. It highlighted that the *ex situ* approach (where soils were excavated, aerated and fertilized in an adjacent biopile to specifically stimulate aerobic bacteria) had a larger and more consistent influence on the microbial community structure and activities than the *in situ* approach (where soils were fertilized in place to keep soil structure intact) and resulted therefore in higher rates of hydrocarbon degradation. In the *ex situ* biopile experiment, located at Alert, Nunavut, Canada, a clear reorganization of the microbial community and a large increase in the expression of hydrocarbon degrading genes were observed one month after starting the treatment. However, information is still missing as to which microorganisms and which functional genes are associated with bioremediation experiments having high degradation rates, like the one at Alert. This gap in knowledge hampers the design of bioremediation strategies targeting specific microorganisms associated with high degradation rates. A targeted approach could lead to more rapid bioremediation, an important factor considering that ambient temperatures are above freezing for less than 2 months during the Arctic summer. The microorganisms and functional genes associated with high hydrocarbon degradation rates could also be useful indicators of the potential of soils for hydrocarbon bioremediation and could be interesting model organisms to study cold temperature bioremediation and as a source of cold-adapted enzymes.

Microbial degradation of complex hydrocarbon mixtures, such as diesel, requires several different genes and pathways. Diesel fuel is composed of both saturated aliphatic (alkanes) and aromatic hydrocarbons. We therefore focused our data mining effort on alkane hydroxylases and aromatic-ring-cleavage dioxygenase genes. Hydroxylation of an alkyl group catalyzed by oxygenases is usually the first step in the degradation of organic compounds. There are several categories of alkyl-group hydroxylases, including cytochrome P450s (CYP) and alkane hydroxylase [14]. The alkane hydroxylase catalyzes the hydroxylation of the terminal carbon of alkanes and consists of three different subunits, including the membrane-bound hydroxylase subunit encoded by *alkB*. CYP153, an enzyme of the CYP superfamily, can also catalyze the hydroxylation of alkanes [15]. Aromatic rings also need to be hydroxylated to be degraded, but the key step in aromatic hydrocarbon degradation is the opening of the hydroxylated aromatic ring, which is catalyzed by aromatic-ring-cleavage dioxygenases [14]. There are three main types of aromatic-ring-cleavage dioxygenases (intradiol, extradiol and gentisate/homogentisate) that can be differentiated based on their substrate and on the position where the ring fission occurs relative to the hydroxyl groups [14].

The main goal of this study was to monitor the microbial communities in Alert biopiles over time to identify microorganisms and functional genes linked to the high hydrocarbon degradation rates previously observed in these soils undergoing treatment. In order to accomplish this goal, we needed an unbiased, culture- and PCR-independent method that could yield insights into community composition and functional potential at the same time. We therefore sequenced the metagenome of Alert soil biopiles through a time course and compared results with uncontaminated soil. We also quantified the expression and the abundance of key functional genes for abundant microorganisms identified in the metagenomic datasets. From our results, we concluded that hydrocarbon contamination and biopile treatment (e.g. excavation, aeration and fertilization) dramatically changed soil microbial communities, favoring aerobic organisms that have the potential to degrade various hydrocarbon compounds (e.g. *Pseudomonas*, *Rhodococcus*, *Sphingomonas*, *Caulobacter*). The relative dominance of these bacteria varied over time, likely a result of changes in the quality and availability of various hydrocarbons and nutrients during bioremediation.

## Materials and Methods

### Ethics statement

No specific permits were required for the described field studies at Alert. The study location is not privately owned or protected in any way and the field studies did not involve endangered or protected species.

### Site description, bioremediation treatments and soil sampling

For the present study, we analyzed a subset of the samples used previously in Yergeau and colleagues [13]. These samples were taken from a bioremediation experiment at Alert (82°31′ N, 62°17′ W), Ellesmere Island, Nunavut, in the Canadian high Arctic. The soil under study was impacted by a large diesel spill in 2004 and used to construct biopiles in 2005, which were aerated and fertilized with NH_4_PO_4_ (monoammonium phosphate, MAP) to specifically stimulate aerobic microorganisms. An uncontaminated control soil was collected from an area directly adjacent to the original spill site at a depth of approximately 30 cm, but was not excavated, aerated or fertilized. It is representative of the soil conditions before excavation or contamination by hydrocarbons. More details about the experimental design and the site characteristics were previously published [13]. Two samples were collected from the contaminated soil after excavation, but before biopile construction and nutrient amendment (t = 0, August 2005), 4 samples were taken one month after nutrient amendment and mixing (t = 1m, September 2005), 4 samples were taken one year after nutrient amendment (t = 1y, August 2006) and 1 uncontaminated control soil sample was collected adjacent to the original spill site (August 2005). Soil samples were composites (each composed of 5 sub-samples) collected throughout the biopiles. All replicates were used for soil analyses, mineralization assays, qPCR and RT-qPCR assays while replicates were pooled for metagenomic sequencing. More details about soil sampling are available in Yergeau and colleagues [13].

### Soil analyses and mineralization assays

Soil analyses and mineralization assays were previously described and published in Yergeau and colleagues [13]. Briefly, soil petroleum hydrocarbons (C10-C50 or F1–F4 fractions) were assessed by gas chromatography/flame ionization detector (GC/FID). Potential hexadecane and naphthalene mineralization was assessed at 4°C in the laboratory following Whyte and colleagues [16]. Soil hydrocarbon concentration decreased rapidly 1 month after the beginning of the treatment (−59%) and had almost disappeared 1 year after treatment began (−91%) [13]. Values were significantly different for each sampling date and for the uncontaminated soil [13]. Potential naphthalene mineralization at 4°C did not vary significantly along the bioremediation time course, but contaminated samples showed higher mineralization activity than uncontaminated samples [13]. Potential hexadecane mineralization at 4°C was significantly higher in t = 1m samples than all other samples, and significantly higher in t = 1y samples than in t = 0 and uncontaminated samples [13]. Potential naphthalene mineralization rates were higher and more significant than potential hexadecane mineralization rates for all samples.

### Nucleic acid extraction

Soil DNA was extracted from 0.5 g soil sub-samples using the MoBio DNA Power Soil kit (MoBio Laboratories, Carlsbad, CA), while soil RNA was extracted from a 2.0 g soil sub-sample using the MoBio RNA Power Soil kit. Residual DNA in RNA extracts was removed using the Turbo DNA-*free* kit (Ambion, Austin, TX). Average DNA yields were: t = 0: 445.4 ng; t = 1m: 1248.2 ng; t = 1y: 1433.9 ng; uncontaminated: 834.4 ng. Average RNA yields were (after digestion): t = 0: 781 ng; t = 1m: 6110 ng; t = 1y: 4966ng; uncontaminated: 2289 ng.

### Reverse-transcriptase real-time PCR (RT-qPCR) and real-time PCR (qPCR)

RT-qPCR was performed in 20 µl volumes using the iScript One-Step RT-PCR kit with Sybr green (Bio-Rad Laboratories, Hercules, CA) on a Rotor-Gene 3000 apparatus (Corbett Life Science, Sydney, Australia), as previously described [13], [17]. Reactions were set-up as per the manufacturer's instructions, with 1–75 ng of total soil RNA. The amplification procedure was as follows: cDNA synthesis for 10 min at 50°C, reverse transcriptase inactivation for 5 min at 95°C, PCR cycling and detection (40 cycles) for 10 s at 95°C, 15 s at 57°C and 15 s at 72°C (acquiring signal at the end of this step). Primers were designed to specifically match the *alkB* and *ndoB* genes of *Pseudomonas* strains and the *alkB1* and *alkB2* genes of *Rhodococcus* strains previously isolated from high Arctic contaminated soils [4], [16]. The primers used were (5′→3′): *Pseudomonas alkB*: *alkB*(PspEu5)qF1 (GGG CTT GAG GAA CAA CG) with *alkB*(PspEu5)qR1 (CAC CAM CCG AAA GCC AT), *Rhodococcus alkB1*: *alkB1*(Q15)qF1 (GAT TTG AGC GTT CTC TCC AAT A) with *alkB1*(Q15)qR2 (TCG AGG TAG AAG TGA CCG TAA G), *Rhodococcus alkB2*: *alkB2*-Rh-F (CCT GGC TCG GAA TCG AC) with *alkB2*-Rh-R (GAA GTG GCC GTA AAA CGA CT) and *Pseudomonas ndoB*: *ndoB*-Ps-F (CTC CAA CGG TGA ACT GCA) with *ndoB*-Ps-R (CAA CCT TGC CTG GAG GAC). Although these primers were designed some time ago, their specificity was re-examined by blasting them against NCBI-nr and looking at *alkB* sequences that perfectly matched both primers. The majority of the hits for the *Pseudomonas alkB* primers were related to a variety of *Pseudomonas* species, but several hits to other genera were also observed. All these hits were identical to *Pseudomonas alkB* genes over 870 bp and were all produced in the same study [18] that indicated that this high similarity might be due to horizontal gene transfer. The *Rhodococcus* sp. *alkB1* and *alkB2* primers matched a variety of *Rhodococcus* sequences but also the *alkB* genes of three other species that shared 98–99% sequence similarity with *Rhodococcus*. The *Pseudomonas ndoB* primers perfectly matched a variety of *Pseudomonas* species and no other species. For all the non-specific hits mentioned above, the bacteria associated with the genes were either not retrieved or retrieved at very low abundance in the metagenomic datasets. Standards were made from 10-fold dilutions of linearized plasmids containing the gene fragment of interest cloned from DNA amplified from pure strains. For all reactions, several no-reverse-transcriptase and no-template controls were included and yielded no detectable signals.

qPCR were performed in 20 µl volumes using the QuantiTect SYBR Green PCR kit (Qiagen, Mississauga, ON, Canada) on a Rotor-Gene 3000 apparatus (Corbett Life Science, Sydney, Australia). Reactions were set-up as per the manufacturer's instructions, with 1.5–10 ng of total soil DNA. The qPCR conditions, primers and standards were as in the RT-qPCR runs, except that the reverse transcription step was eliminated, the initial denaturation was extended to 15 min and the annealing temperature was raised to 60°C. Lambda DNA was used to correct for potential PCR inhibitors present in soil DNA and RNA extracts [19]. Equal volumes of soil RNA or DNA extracts and a cloned 500-bp fragment of bacteriophage lambda (10^5^ copies per µl) were mixed. When the recovery of lambda in RT-qPCR and qPCR reactions was below 100%, quantification values for all other genes were corrected accordingly. PCR inhibition ranged from 6.2% to 45.1% (RNA) and from 0.0% to 51.4% (DNA).

### Metagenomic sequencing and bioinformatic analyses

At the time of sequencing, the amount of DNA needed for 454 shotgun library preparation was 5 µg. Since Arctic soils have relatively low biomass, we could not retrieve such large amounts from our DNA extractions. For that reason, we pooled the different biological replicates before metagenomic sequencing. This also maximized the depth of sequencing within the limits of the financial resources available. This decision was further supported by the fact that 16S rRNA and hydrocarbon-degrading gene microarray analyses previously showed that within treatment (i.e. same sampling times) variation was small compared to between treatment (i.e. different sampling times) variation [13]. We used PermANOVA (adonis function in R) to statistically evaluate if the within treatment variation was significantly lower than the between treatment variation and found out that it was for both the 16S rRNA gene microarray (F = 0.843, *P* = 0.001) and the hydrocarbon-degrading gene microarray (F = 0.612, *P* = 0.007). We therefore sequenced four DNA samples: one composed of samples taken before the bioremediation treatments (t = 0), one composed of samples taken one month after the beginning of the treatments (t = 1m), one composed of samples taken one year after the beginning of the treatments (t = 1y) and one control sample that was uncontaminated. DNA from the different treatments was sent for two runs of Roche 454 GS FLX Titanium sequencing at The Centre for Applied Genomics, The Hospital for Sick Children, Toronto, Canada.

Data analyses were essentially carried out as previously described [20]. Replicated sequences were removed from the dataset using the method of Gomez-Alvarez and colleagues [21]. After this step, we had four datasets with the following characteristics: t = 0: 364,725 sequences of 343 bp (on average) for a total of 125 Mbp; t = 1m: 108,203 sequences of 327 bp (on average) for a total of 35 Mbp; t = 1y: 287,705 sequences of 350 bp (on average) for a total of 101 Mbp; t = 0: 457,781 sequences of 441 bp (on average) for a total of 202 Mbp. Sequences were then annotated using the MG-RAST v 2.0 web service [22]. The maximum E-value for a significant match was set to 10^−5^ and the minimum alignment length was set to 50 bp. Taxonomic profiles based on all annotated fragments were normalized by dividing the number of fragments matching an organism by its genome size to correct for the fact that, for the same abundance, organisms with larger genomes will have more hits than organisms with smaller genomes. Taxonomic profiles were also made using the fragments matching 16S rRNA genes. These sequences were retrieved from MG-RAST and classified to the genus level using the “Classifier” tool of the Ribosomal Database Project (RDP, http://rdp.cme.msu.edu/) with a bootstrap value of 50% (which is the level recommended by RDP for short sequences). For the 16S rRNA gene taxonomic profiles to be comparable with the taxonomic profiles based on all fragments, only sequences that could be classified at the phylum level were used.

Since most hydrocarbon-degrading genes are not clearly annotated in the SEED and are thus not classified in MG-RAST subsystems, we used BLAST to fish out all the major genes involved in hydrocarbon degradation from the datasets. Each individual metagenomic dataset was used as a BLAST database against which reference protein sequences from one Gram-positive and one Gram-negative species were compared (when available). The reference sequences used were (EC number; SwissProt accessions): alkane-1 monooxygenase protein (EC 1.14.15.3; O05895 and P12691), cytochrome P450 alkane hydroxylase (CYP153) protein (EC 1.14.-.-; Q2MHE2 and A9CMS7), protocatechuate 3,4-dioxygenase (EC 1.13.11.3; alpha and beta subunits: P20371, P20372, Q8NN16 and Q8NN15), catechol 1,2-dioxygenase (EC 1.13.11.1; P95607 and P07773), gentisate 1,2-dioxygenase (EC 1.12.11.4; Q13ZY3 and Q0SFK9), homogentisate 1,2-dioxygenase (EC 1.13.11.5; B8H072 and Q828S5), catechol 2,3-dioxygenase (EC 1.13.11.2; P06622 and Q53034), protocatechuate 4,5-dioxygenase (EC 1.13.11.8; P22635). All hits having an E-value below 0.10 were considered as significant and the corresponding sequences were recruited from the metagenomic datasets. We used a very low E-value for recruiting sequences, to not miss any sequences that might be from distantly related organisms. This procedure allowed our analysis to be as inclusive as possible. The recruited sequences were then subjected to blastx against the NCBI non-redundant protein sequences (nr) and the best match was kept to determine the taxonomic affiliation of the recruited sequence. Sequences recruited from our datasets using reference catechol 2,3-dioxygenase sequences also matched sequences related to biphenyl-2,3-diol 1,2-dioxygenase (EC 1.13.11.39) or 1,2-dihydroxynaphthalene dioxygenase (EC 1.13.11.-), which are closely related, and these sequences were kept. In all other cases, the recruited sequences not clearly related to the proteins of interest were discarded. We determined that sequences recruited with E-values above 10^−30^ generally did not match the protein of interest in subsequent blast searches, indicating that our procedure recruited all sequences related to the gene of interest. The number of hits was summed for each genus and normalized per 100 genomes (3Mbp genomes; 300Mbp equivalent to 750,000 reads of 400 bp) to account for differences in dataset sizes. For comparison purposes, normalization was also performed by randomly selecting 100,000 sequences from each of the datasets and re-running the BLAST analyses. The two normalization procedures provided highly similar results (Table 1 vs. Tables 2–5), and values were highly correlated (r_s_ = 0.911, *P*<0.00001).

**Table 1:** Number of sequences having significant matches with functional genes (e-value<10^−5^) in normalized datasets (100,000 sequences).

	uncont.	t = 0	t = 1m	t = 1y
**Alkane hydroxylase**	4	31	32	41
**Cytochrome P450**	35	41	48	62
**Extradiol**				
Catechol 2,3-dioxygenase	1	20	18	20
Protocatechuate 4,5-dioxygenase	1	2	1	1
**total**	2	22	19	21
**Intradiol**				
Catechol 1,2-dioxygenase	5	43	13	20
Protocatechuate 3,4-dioxygenase	21	48	19	17
**Total**	26	91	32	37
**Gentisate/Homogentisate**				
Gentisate 1,2-dioxygenase	10	14	13	14
Homogentisate 1,2-dioxygenase	18	56	4	23
**Total**	28	70	17	37

Values are normalized by randomly sampling 100,000 sequences per sample.

**Table 2:** Taxonomic affiliation and relative abundance of alkyl group hydroxylase related sequences retrieved in the metagenomic datasets.

Phylum/class	Uncont.	t = 0	t = 1m	t = 1y
**Alkane hydroxylase (EC 1.14.15.3)**				
*Actinobacteria*	3.2	24.8	6.9	57.3
*Alphaproteobacteria*	4.8	24.8	0.0	36.5
*Betaproteobacteria*	0.0	2.1	0.0	0.0
*Gammaproteobacteria*	1.6	45.3	62.4	18.2
*Firmicutes*	0.0	0.0	0.0	2.6
*Eukaryotes*	0.0	0.0	6.9	0.0
synthetic construct	0.0	2.1	13.9	2.6
uncultured/unclassified	1.6	14.4	27.7	10.4
**Total**	11.2	113.5	117.8	127.6
**Cytochrome P450 (EC 1.14.-.-)**				
*Acidobacteria*	1.6	0	0	0
*Actinobacteria*	55.6	105	103.9	130
*Alphaproteobacteria*	42.4	54	13.8	52
*Betaproteobacteria*	3.2	8.3	0	2.6
*Gammaproteobacteria*	1.6	10	6.9	10.4
*Deltaproteobacteria*	9.8	4.1	0	0
*Chloroflexi*	9.9	0	6.9	0
*Firmicutes*	3.3	0	0	0
*Planctomycetes*	0	0	0	2.6
Eukaryotes	0	2.1	0	0
Uncultured/unclassified	4.9	12	13.9	36.5
**Total**	132.3	196	145.4	234

Values are normalized per 100 genomes (assuming 3Mbp genomes and 400 bp reads).

**Table 3 pone-0030058-t003:** Taxonomic affiliation and relative abundance of extradiol aromatic-ring-cleavage dioxygenase related sequences retrieved in the metagenomic datasets.

Phylum/class	Uncont.	t = 0	t = 1m	t = 1y
**Catechol 2,3-dioxygenase (EC 1.13.11.2)**				
*Actinobacteria*	3.3	2.1	6.9	5.2
*Alphaproteobacteria*	1.6	10.3	0.0	5.2
*Betaproteobacteria*	0.0	2.1	0.0	13.0
*Gammaproteobacteria*	0.0	4.1	20.8	0.0
*Chloroflexi*	1.6	0.0	0.0	0.0
*Deinococcus-Thermus*	3.2	0.0	0.0	0.0
*Firmicutes*	4.8	0.0	6.9	2.6
uncultured/unclassified	4.9	18.5	13.9	13.0
Total	19.4	37.1	48.5	39.0
**Biphenyl-2,3-diol 1,2-dioxygenase (EC 1.13.11.39)**				
*Actinobacteria*	1.6	20.7	6.9	15.6
*Alphaproteobacteria*	4.9	10.4	0.0	10.4
*Betaproteobacteria*	1.6	2.1	6.9	2.6
*Gammaproteobacteria*	1.6	4.1	0.0	5.2
uncultured/unclassified	0.0	2.1	6.9	0.0
Total	9.7	39.4	20.7	33.8
**1,2-dihydroxynaphthalene dioxygenase (EC 1.13.11.-)**				
*Alphaproteobacteria*	0.0	2.1	0.0	0.0
*Gammaproteobacteria*	0.0	2.1	0.0	0.0
Total	0.0	4.2	0.0	0.0
**Protocatechuate 4,5-dioxygenase (EC 1.13.11.8)**				
*Actinobacteria*	1.6	6.2	0.0	0.0
*Alphaproteobacteria*	0.0	10.4	6.9	2.6
*Betaproteobacteria*	0.0	10.4	0.0	2.6
*Gammaproteobacteria*	1.6	4.2	0.0	0.0
Total	3.2	31.2	6.9	5.2
**Total extradiol**	32.3	111.9	76.1	78.0

Values are normalized per 100 genomes (assuming 3Mbp genomes and 400 bp reads).

**Table 4 pone-0030058-t004:** Taxonomic affiliation and relative abundance of intradiol aromatic-ring-cleavage dioxygenase related sequences retrieved in the metagenomic datasets.

Phylum/class	Uncont.	t = 0	t = 1m	t = 1y
**Catechol 1,2-dioxygenase (EC 1.13.11.1)**				
*Actinobacteria*	3.3	14.5	6.9	7.8
*Alphaproteobacteria*	1.6	14.5	6.9	13.0
*Betaproteobacteria*	3.2	12.6	0.0	18.2
*Gammaproteobacteria*	1.6	24.8	13.8	13.0
*Bacteroidetes*	1.6	0.0	0.0	0.0
uncultured/unclassified	0.0	6.2	0.0	5.2
Total	11.3	72.6	27.6	57.2
**Protocatechuate 3,4-dioxygenase (EC 1.13.11.3)**				
*Actinobacteria*	9.6	22.7	34.5	7.8
*Alphaproteobacteria*	8.0	2.1	6.9	2.6
*Betaproteobacteria*	13.0	4.2	6.9	2.6
*Gammaproteobacteria*	8.2	30.8	27.7	5.2
*Eukaryotes*	0.0	0.0	0.0	2.6
uncultured/unclassified	8.2	0.0	6.9	0.0
Total	47.0	59.8	82.9	20.8
**Total intradiol**	58.3	132.4	110.5	78.0

Values are normalized per 100 genomes (assuming 3Mbp genomes and 400 bp reads).

**Table 5:** Taxonomic affiliation and relative abundance of gentisate/homogentisate aromatic-ring-cleavage dioxygenase related sequences retrieved in the metagenomic datasets.

Genus	uncont.	t = 0	t = 1m	t = 1y
**Gentisate 1,2-dioxygenase (EC 1.13.11.4)**				
*Actinobacteria*	0	6.2	13.8	5.2
*Alphaproteobacteria*	6.5	6.2	0	5.2
*Betaproteobacteria*	14.5	17	13.8	15.6
*Gammaproteobacteria*	4.9	0	0	0
total	25.9	29	27.6	26
**Homogentisate 1,2-dioxygenase (EC 1.13.11.5)**				
*Acidobacteria*	4.9	0	0	2.6
*Actinobacteria*	3.2	2.1	0	0
*Alphaproteobacteria*	13	31	0	33.8
*Betaproteobacteria*	14.6	8.3	6.9	18.2
*Gammaproteobacteria*	8.2	60	13.9	46.9
*Deltaproteobacteria*	1.6	0	0	7.8
*Bacteroidetes*	1.6	4.2	0	0
*Chloroflexi*	4.9	0	0	2.6
*Firmicutes*	3.2	0	0	0
*Gemmatimonadetes*	1.6	0	0	0
Total	56.8	105	20.8	112
**total gentisate/homogentisate**	82.7	134	48.4	138

Values are normalized per 100 genomes (assuming 3Mbp genomes and 400 bp reads).

### Statistical analyses

All statistical analyses were performed in R (version 2.9.0, The R Foundation for Statistical Computing, Vienna, Austria). When necessary, data was log or square root transformed to meet the assumptions of parametric ANOVA. Normality was tested using the “shapiro.test” function. ANOVA and subsequent Tukey HSD tests were carried out using the “aov” and “TukeyHSD” functions, respectively. When transformations failed to normalize data, Kruskal-Wallis and associated multiple comparison tests were carried out using the “kruskal.test” and the “kruskalmc” functions of the “pgirmess” library, respectively. Correlation analyses were based on Spearman's *r* using the “cor” function.

### Data deposition

Metagenomic datasets were deposited in the NCBI sequence read archive (SRA) under accession number SRA025056. The metagenomic project can also be accessed in NCBI under GenomeProject ID 56113 (accession PRJNA56113, http://www.ncbi.nlm.nih.gov/bioproject?term=PRJNA56113).

## Results

### Metagenomic sequencing: taxonomic profile

**Figure 1:**
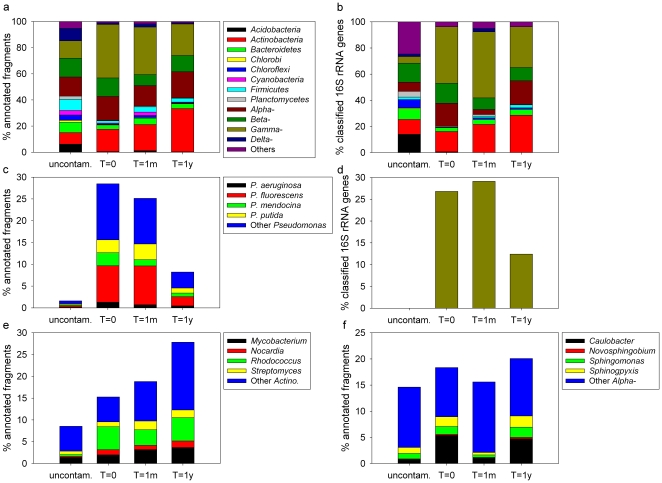
Taxonomic community composition. Bacterial community taxonomic composition based on metagenomic sequencing. (a) Community composition at the phylum/class level for t = 0, t = 1m, t = 1y and uncontaminated Alert biopile soils based on all annotated fragments. “Others” include: *Aquificae*, *Chlamydiae*-*Verrucomicrobia*, *Deinococcus*-*Thermus*, *Epsilonproteobacteria*, *Fusobacteria*, *Spirochaetes*, *Synergistetes*, *Thermotogae* and unclassified Bacteria. (b) Community composition at the phylum/class level for t = 0, t = 1m, t = 1y and uncontaminated Alert biopile soils based on classified 16S rRNA genes retrieved in the metagenomic dataset. “Others” include: *Gemmatimonadetes*, *Nitrospira*, OD1, OP10, *Verrucomicrobia*, and TM7. (c) *Pseudomonas* relative abundance for t = 0, t = 1m, t = 1y and uncontaminated Alert biopile soils based on all annotated fragments. “Others” include: *P. entomophila*, *P. fulva*, *P. resinovorans*, *Pseudomonas* sp. and *P. syringae*. (d) *Pseudomonas* relative abundance for t = 0, t = 1m, t = 1y and uncontaminated Alert biopile soils based on classified 16S rRNA genes retrieved in the metagenomic dataset. (e) *Actinobacteria* relative abundance for t = 0, t = 1m, t = 1y and uncontaminated Alert biopile soils based on all annotated fragments. “Others” include: *Acidothermus*, *Arcanobacterium*, *Bifidobacterium*, *Brevibacterium*, *Clavibacter*, *Corynebacterium*, *Frankia*, *Gordonia*, *Janibacter*, *Kineococcus*, *Leifsonia*, *Micrococcus*, *Propionibacterium*, *Salinispora*, *Thermobifida*, *Tropheryma* and *Rubrobacter*. (f) *Alphaproteobacteria* relative abundance for t = 0, t = 1m, t = 1y and uncontaminated Alert biopile soils based on all annotated fragments. “Others” include: *Acetobacter*, *Acidiphilium*, *Agrobacterium*, *Anaplasma*, *Aurantimonas*, *Bartonella*, *Bradyrhizobium*, *Brucella*, *Dinoroseobacter*, *Ehrlichia*, *Erythrobacter*, *Gluconobacter*, *Granulibacter*, *Hyphomonas*, *Loktanella*, *Magnetospirillum*, *Maricaulis*, *Mesorhizobium*, *Methylobacterium*, *Neorickettsia*, *Nitrobacter*, *Oceanicaulis*, *Parvibaculum*, *Parvularcula*, *Rhizobium*, *Rhodobacter*, *Rhodobacter*, *Rhodopseudomonas*, *Rhodospirillum*, *Rickettsia*, *Roseobacter*, *Roseovarius*, *Ruegeria*, *Silicibacter*, *Sinorhizobium*, *Sulfitobacter*, *Wolbachia*, *Zymomonas*.

Contamination and soil excavation resulted in the increase of the abundance of several bacterial phylum/classes, like the *Actinobacteria*, the *Alphaproteobacteria* and the *Gammaproteobacteria* (Fig. 1a, 1b). In contrast, contamination and excavation resulted in a decrease in the abundance of *Acidobacteria*, *Bacteroidetes*, *Chlorobi*, *Chloroflexi*, *Cyanobacteria*, *Firmicutes*, *Planctomycetes* and *Deltaproteobacteria* (Fig. 1a, 1b). The contaminated samples (t = 0, t = 1m and t = 1y) showed relatively similar taxonomic profiles at the phylum/class level, but with some interesting differences. The *Actinobacteria* increased through the time course while the *Gammaproteobacteria* decreased (Fig. 1a, 1b). In the t = 0 and t = 1m samples, *Gammaproteobacteria* comprised up to ∼30% of the total community. At the phylum/class level, the uncontaminated sample showed a diversified taxonomic profile, with no clear dominance of any of the phyla/classes. The high abundance of *Gammaproteobacteria* in the t = 0 and t = 1y samples was mainly due to *Pseudomonas* species (Fig. 1c, 1d). The relative abundance of *Pseudomonas* decreased substantially at t = 1y and was very low or undetected in the uncontaminated samples. Among the *Actinobacteria*, *Mycobacterium* and *Streptomyces* increased through the time course, while *Rhodococcus* and *Nocardia* decreased from t = 0 to t = 1m and then increased from t = 1m to t = 1y (Fig. 1e). For the *Alphaproteobacteria*, *Caulobacter* and the sphingomonads had similar abundances at t = 0 and t = 1y, but lower abundances at t = 1m (Fig. 1f). Among the genomes available in the MG-RAST, the strains onto which the highest numbers of fragments mapped were *Pseudomonas fluorescens* PfO-1 (t = 0 and t =  1m), marine *Actinobacterium* PHSC20C1 (t = 1y) and the Acidobacteria *Solibacter usitatus* Ellin6076 (uncontaminated). The genera that dominated the retrieved 16S rRNA gene fragments were *Pseudomonas* (t = 0, t = 1m and t = 1y) and an unclassified GP4 *Acidobacteria* (uncontaminated).

### Metagenomic sequencing: functional profile

The general high-level functional profiles of the samples based on the MG-RAST subsystems (subsystem hierarchy 1) were very similar. Functional profiles were also very similar at deeper levels within the subsystem hierarchy (Subsystem hierarchy 2 and Subsystems; not shown). In contrast, there were large changes in the abundance and taxonomic affiliation of the genes encoding enzymes responsible for diesel degradation (alkane hydroxylase, CYP and aromatic-ring-cleavage dioxygenases) through the time course (Tables 2–5). The number of alkane hydroxylase related sequences (*alkB*; normalized per 100 genomes) was stable over the time course, but ten times lower in the uncontaminated sample (Table 2). The t = 0 and t = 1m *alkB*-related sequences were mostly associated with the *Gammaproteobacteria*, with a significant proportion also being associated with the *Alphaproteobacteria* and the *Actinobacteria* in the t = 0 sample (Table 2). In the t = 1y sample, most of the *alkB* sequences were related to the *Alphaproteobacteria* and the *Actinobacteria*, and the sequences related to the *Gammaproteobacteria* only formed a relatively minor part of the *alkB* sequences. In the uncontaminated sample, *alkB* genes were related to a variety of genera, with no clear dominance. The number of cytochrome P450 (CYP) genes (normalized per 100 genomes) was similar in the t = 0 and t = 1m samples, but higher in the t = 1y samples and slightly lower in the uncontaminated sample (Table 2). In all the contaminated samples (t = 0, t = 1m and t = 1y), the dominant CYP sequence type matched the *Actinobacteria* with a significant proportion matching the *Alphaproteobacteria* (Table 2).

Extradiol aromatic-ring-cleavage dioxygenase (EARCD) sequences were more abundant in the t = 0 sample (Table 3). This was mainly due to the much higher relative abundance and diversity of protocatechuate 4,5-dioxygenase in the t = 0 sample as compared to all other samples (Table 3). All the other EARCD genes were similarly abundant in the contaminated soils, but less abundant in the uncontaminated sample. For the EARCD, no clear dominance by phylum/class was observed and a variety of phyla/classes were the most abundant, depending on the dioxygenase type/sample, and there was also a significant number of matches to uncultured/unclassified organisms for the catechol 2,3-dioxygenase (Table 3). The number of intradiol aromatic-ring-cleavage dioxygenase (IARCD) genes (normalized per 100 genomes) was higher in the contaminated soil as compared to the uncontaminated soil (Table 4). More specifically, the relative abundance of catechol 1,2-dioxygenase was higher in the t = 0 and t = 1y samples and very low in the uncontaminated sample, while the relative abundance of protocatechuate 3,4-dioxygenase was higher in the t = 1m sample and relatively high in the uncontaminated sample (Table 4). The *Gammaproteobacteria* was generally the main phylum/class linked to IARCD sequences in contaminated samples, with some exceptions where the *Actinobacteria* or the *Betaproteobacteria* were the most frequently observed phylum/class (Tables 4). Gentisate 1,2-dioxygenase related sequences were similarly abundant in all samples (Table 5). The *Betaproteobacteria* was the dominant phylum/class related to gentisate 1,2-dioxygenase (Table 5). In contrast, homogentisate 1,2-dioxygenase related sequences were more abundant in the t = 0 and t = 1y samples (Table 5). The most abundant phylum/class related to homogentisate 1,2-dioxygenase sequences was the *Gammaproteobacteria* in the contaminated samples, while it was the *Betaproteobacteria* for the uncontaminated sample (Table 5).

### qPCR and RT-qPCR

**Figure 2:**
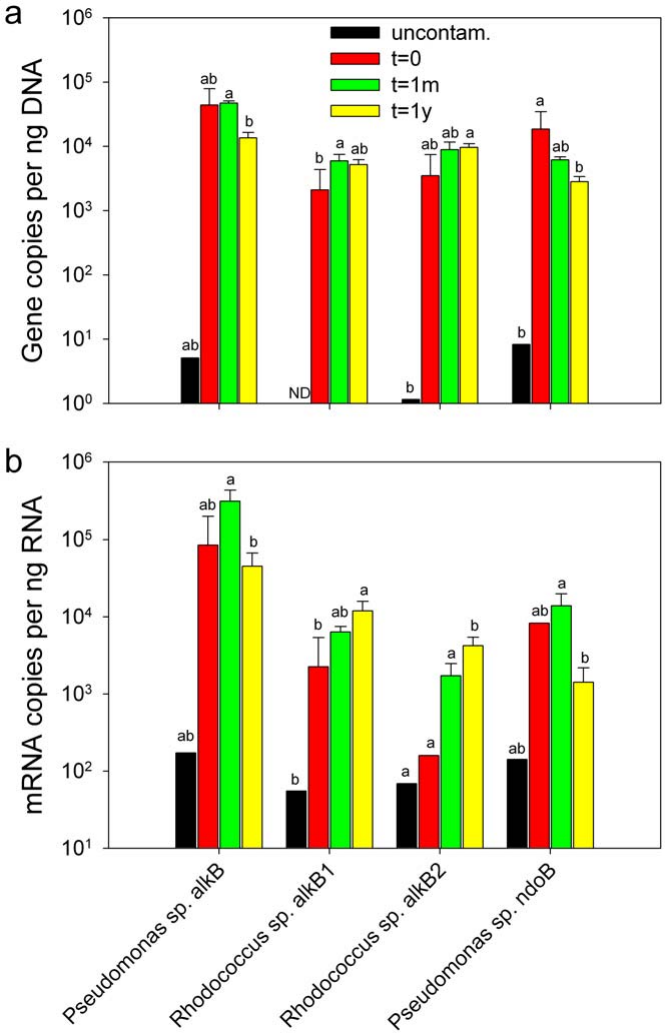
Hydrocarbon-degrading gene relative abundance and expression. *Pseudomonas alkB* and *ndoB* and *Rhodococcus alkB1* and *alkB2* genes (a) relative abundance and (b) relative expression in t = 0, t = 1m, t = 1y and uncontaminated Alert biopile soils. Different letters within a group of bars (e.g. *Pseudomonas* sp. *alkB*) mean significantly different averages (P<0.05) (For t = 0, N = 2; for t = 1m, N = 4; for t = 1y N = 4; for uncontaminated, N = 1).

We quantified the abundance and the expression of four genes in all replicates: *Pseudomonas alkB* and *ndoB* genes and *Rhodococcus alkB1* and *alkB2* genes (Fig. 2). The number of gene copies was very low for all genes in the uncontaminated sample (Fig. 2a). The *Pseudomonas* genes (*alkB* and *ndoB*) showed a decrease through the time course, with their abundance being significantly lower in the t = 1y samples, compared to the t = 1m (for the *alkB*) or the t = 0 (for the *ndoB*) samples. In contrast, the abundance of the *Rhodococcus alkB* genes was usually lower in the t = 0 samples, but this difference was not always significant. There were significant correlations between the soil hydrocarbon content and the abundance of *Pseudomonas alkB* and *ndoB* genes (r_s_ = 0.782, *P* = 0.00653 and r_s_ = 0.891, *P* = 0.00322, respectively).

The expression of the same genes measured by RT-qPCR followed a somewhat different pattern (Fig. 2b). The expression of *Pseudomonas alkB* and *ndoB* was higher in the t = 1m samples. In contrast, *Rhodococcus alkB* genes were more expressed in the t = 1y samples.The expression of all genes was very low in the uncontaminated samples. There was a significant correlation between hexadecane mineralization and the expression of *Pseudomonas alkB* genes (r_s_ = 0.809, *P* = 0.00407). Based on total *alkB* RT-qPCR quantification of a previous study using the same samples, which were 1.26×10^6^ (t = 0), 2.26×10^6^ (t = 1m), 1.72×10^5^ (t = 1y) and 3.89×10^2^ (uncontaminated) copies per ng of RNA [13], *Pseudomonas* sp. *alkB* gene expression represented 16.07%, 13.37%, 27.91% and 44.38% of the total *alkB* gene expression for samples t = 0, t = 1m, t = 1y and uncontaminated, respectively. Similarly, *Rhodococcus* sp. *alkB* gene expression (sum of *alkB1* and *alkB2* gene expression) represented 0.46%, 0.38%, 16.59% and 31.80% of the total *alkB* gene expression for samples t = 0, t = 1m, t = 1y and uncontaminated, respectively.

## Discussion

A previous study from our group showed that, as compared to other high Arctic bioremediation experiments, Alert soils had very high hydrocarbon degradation rate [13]. This elevated degradation rate was linked to major shifts in bacterial community structure (based on microarray analyses) and to increases in the total expression of different functional genes (RT-qPCR of *alkB* and polyaromatic hydrocarbon ring hydroxylating dioxygenase genes). However, the methods used in this previous study did not allow us to identify the microorganisms that might be involved in hydrocarbon degradation. We therefore used shotgun metagenomic sequencing, a method that is unbiased, culture- and PCR-independent, to reveal the main hydrocarbon degraders present in these soils showing high degradation rates. Based on the results of metagenomic sequencing, the expression and the abundance of key functional genes for two of the most dominant microorganisms were quantified.

### Community composition

The largest differences in bacterial communities were observed between the uncontaminated, undisturbed soil and the contaminated biopile soils. One of the keys to successful and rapid bioremediation is to specifically stimulate the fast-growing, hydrocarbon-degrading bacteria that are already present in the soils, and such a dramatic shift was therefore expected and desired. Well-known hydrocarbon degraders like *Pseudomonas*, *Rhodococcus*, *Caulobacter* and sphingomonads appeared to be enriched in well-aerated soils with larger amounts of nutrients (N) and carbon (diesel) as compared to intact uncontaminated soils. In the early stage samples (t = 0 and t = 1m), a large number of the identified metagenomic fragments (>25% in the t = 0 sample) could be mapped to *Pseudomonas* species. A previous study showed that *Pseudomonas* is enriched following contamination events [23] and accordingly, we found a positive correlation between the abundance of *Pseudomonas* hydrocarbon degrading genes (by qPCR) and soil hydrocarbon content. *Pseudomonas* species were hypothesized to be one of the major alkane and aromatic hydrocarbon degraders in polar soils [6], [23]. Previous comparisons of Alert soils with other soils located on Ellesmere Island using microarrays showed that *Pseudomonas* genes were more often detected in Alert soils as compared to the other soils [13]. Several hydrocarbon-degrading *Pseudomonas* species are psychrotolerant [4], and this could give them a competitive advantage over mesophilic and psychrophilic species that can only grow over a narrow range of temperatures. It appears that *Pseudomonas* species are taking advantage of their ability to degrade various hydrocarbons and their capacity to grow rapidly at low temperatures [4] to dominate the bacterial communities following diesel contamination of soil and in the early stages of the bioremediation process.

The other major hydrocarbon-degrading genera detected could also have important roles in bioremediation, especially in the later steps of the bioremediation process (i.e. in the t = 1m and t = 1y samples), where they reach their maximum abundance and *Pseudomonas* abundance decreases. For instance, *Alphaproteobacteria* were among the most abundant groups of polyaromatic hydrocarbon degraders. Together with *Pseudomonas*, the *Alphaproteobacteria* sphingomonads were reported as the typical aromatic-degrading bacteria isolated from polar soils [6], [24]. A recent in situ ^15^N monoammonium phosphate DNA-SIP experiment in high Arctic diesel-contaminated soils showed that, after one month of incubation, the family of bacteria most actively incorporating ^15^N in its DNA was *Sphingomonadaceae*, suggesting an important role for this group of bacteria in the degradation of hydrocarbons in high Arctic soils [25]. Sphingomonads might be more competitive at low nutrient availability or in less well aerated soils, as they were not strongly enriched following contamination and bioremediation treatment. In fact, a strain of *Sphingomonas* isolated from Antarctic soils was shown to degrade the aromatic fraction of diesel at low temperature without nutrient amendment [26]. The *Alphaproteobacteria* had an intriguing presence among the alkane degraders, as they are not reported as typical alkane degraders in polar soils. *Caulobacter* species have the capacity to degrade alkanes, but are more frequently associated with heavy metal resistance [27], [28]. However, *Caulobacter* are typically found in low nutrient environments and some species were reported to be resistant to freezing [29]. *Caulobacter* species were also isolated as part of a chlorophenol-degrading community in cold groundwater [30].

*Rhodococcus* was previously hypothesized to be one of the predominant alkane degrading genera in polar soils [23]. Accordingly, we found here that *Actinobacteria* was the most abundant phylum associated with cytochrome P450 alkane hydroxylases genes in contaminated soils. The presence of cytochrome P450 alkane hydroxylases has been previously linked to an expanded hydrocarbon degradation capacity [31], which might confer a selective advantage to *Actinobacteria* in diesel-contaminated soils especially in the later steps of the bioremediation process. *Rhodococcus alkB* genes were also highly present and active, especially in the t = 1y samples. In fact, *Rhodococcus* relative abundance decreased between t = 0 and t = 1m, but then increased to its maximum value between t = 1m and t = 1y. However, *Rhodococcus* species were not very abundant in the metagenomic data based both on total sequences (0.42% to 5.35% of total reads) and on 16S rRNA gene-related sequences (0% to 5.64% of total 16S rRNA genes). More research would be needed to clearly define the role of *Alphaproteobacteria* and *Actinobacteria* species and their interaction in cold temperature soil hydrocarbon degradation.

The uncontaminated control soil was used to help determine which microorganisms were stimulated by the hydrocarbon contamination and subsequent biopile treatment. Several bacterial phyla/classes showed a preference for uncontaminated soils, having lower abundance in contaminated soils (e.g. *Acidobacteria*, *Planctomycetes*, *Deltaproteobacteria*, *Chlorobi* and *Firmicutes*). This resulted in a higher taxonomic diversity in the uncontaminated sample. Interestingly, uncontaminated soils at Alert appear to be a good source of hydrocarbon degrading bacteria, as we observed several bacteria and functional genes that are involved in hydrocarbon degradation in these soils. Although they are usually present in small numbers in uncontaminated soils, these bacteria can increase and even become dominant following contamination events. For instance, *Pseudomonas*, *Rhodococcus* and *Caulobacter* species were present at very low abundance in uncontaminated soils, but became more abundant in the contaminated soils and during the bioremediation treatment. The fast-growing species that are stimulated by the bioremediation treatment may be less able to compete in undisturbed soil environments with other bacterial taxa that may be better adapted to low levels of carbon, oxygen and nutrients [32].

### High abundance and expression of hydrocarbon degrading genes

We observed a very high relative abundance of genes encoding for hydrocarbon-degrading enzymes, especially in the contaminated samples. In at least one of the samples, for all the gene categories that were mined, the total of genes encoding hydrocarbon-degrading enzymes was above 100 per 100 genome equivalents, indicating that either a large majority of bacteria had one such gene, or that these genes were present in multiple copies in several bacteria. Some of these genes were naturally very abundant in Alert soils (e.g. gentisate dioxygenase) since they were also present in high numbers in uncontaminated samples. Others were highly enriched in contaminated soils (e.g. alkane hydroxylase), which indicate that hydrocarbon contamination and biopile treatment is selecting for microorganisms having the capacity to degrade hydrocarbons.

Using real-time PCR, we confirmed that *Pseudomonas* and *Rhodococcus* species were actively expressing alkane hydroxylase and naphthalene dioxygenase genes. This was important to confirm in the context of this study since metagenomic data only gives an indication of the genetic potential for a function and not whether the gene responsible for this function is actually expressed. *Pseudomonas* was responsible for ∼20% of the total *alkB* expression in soil, which is consistent with its relative abundance. Similarly, *Rhodococcus alkB* expression was consistent with its relative abundance in t = 0 and t = 1m samples. However, *Rhodococcus alkB* relative expression (∼15%) was higher than *Rhodococcus* relative abundance (∼5%) in the t = 1y samples, indicating that *Rhodococcus* were very active in these samples.

### Metagenomics of bioremediation

To the best of our knowledge, the present study represents the first shotgun metagenomic sequencing analysis of a soil bioremediation experiment. This approach is particularly interesting in less-well studied environments like the Canadian high Arctic, since it allows for the discovery of novel genes and organisms that might be missed using traditional PCR- or culture-based techniques. For instance, we reported here that *Caulobacter* could be involved in alkane degradation in Arctic soils, a role that has not been previously reported. We also reported a number of sequences that were related to uncultured microorganisms or that could not be classified, which might represent novel hydrocarbon degradation genes. Furthermore, shotgun metagenomic approaches do not suffer from most of the biases associated with culturing microorganisms and PCR-based methods since it involves direct sequencing of fragmented genomic DNA. Interestingly, the fact that the most abundant bacteria in the t = 0 and t = 1m samples were *Pseudomonas* species indicates that, in this particular case, culture-based methods might have provided valuable information, since *Pseudomonas* species are generally amenable to culture.

However, shotgun metagenomics through 454 sequencing is not completely free of biases, which can still occur during DNA extraction, DNA fragmentation, adaptor ligation, or emulsion PCR. Another more serious drawback of the method used, is that the functional genes screened in this study might be carried on mobile genetic elements, which could decrease the reliability of the taxonomic affiliation made. There is often a lack of congruence between the phylogenies of catabolic genes subjected to horizontal gene transfer (HGT) and 16S rRNA gene phylogenies [33]. Many of the genes selected for deeper analysis in the present study are subject to HGT, being often present on plasmids. A cold-adapted *Pseudomonas* strain isolated from the Canadian high Arctic carried alkane and naphthalene degradation genes on distinct plasmids [4]. Aromatic degradation pathways can also be plasmid-borne, as was shown for a *Sphingomonas* strain [34], [35]. Similarly, several large plasmids involved in straight-chain alkane and aromatic hydrocarbon degradation have been identified in *Rhodococcus* [36]. A linear plasmid was reported to carry three cytochrome P450 genes in a *Rhodococcus* strain [37] which is consistent with the fact that despite the relatively low abundance of *Actinobacteria*, the majority of the CYP450 genes were related to them in our study. In contrast, a cold-adapted strain of *Rhodococcus* did not require any of its two plasmids for alkane mineralization [16]. However, it is still under debate as to what taxonomic extent horizontal gene transfer might occur in natural settings, and there are probably limits to horizontal gene transfer at higher taxonomic levels [38]. Therefore, to improve the robustness of the functional gene analyses carried out, sequences were only classified at the phylum level (or class for *Proteobacteria*). Ideally, the sequences would have been clustered and grouped without prior taxonomic classification [39], but in the context of the shotgun metagenomic study presented here, it is very difficult, if not impossible, to retrieve significant alignments from the fragments sequenced, especially since they are present at relatively low abundance.

### Concluding remarks

Contamination and biopile treatment resulted in clear shifts at both the taxonomic and the functional levels, including an increase in the abundance of several hydrocarbon-degrading genes, suggesting that hydrocarbon contamination and biopile treatment in high Arctic soils selects for a bacterial community that is able to degrade hydrocarbons. Several hydrocarbon-degrading bacteria, like *Pseudomonas*, *Rhodococcus*, *Caulobacter* and sphingomonads, were detected in large numbers in the biopiles and their relative abundance varied over time, probably linked with the variation in hydrocarbon and nutrient quantity and quality.
